# The causes and ecological correlates of head scale asymmetry and fragmentation in a tropical snake

**DOI:** 10.1038/s41598-017-11768-y

**Published:** 2017-09-12

**Authors:** Gregory P. Brown, Thomas Madsen, Sylvain Dubey, Rick Shine

**Affiliations:** 1 School of Life and Environmental Sciences, University of Sydney, NSW 2006 Sydney, Australia; 2 School of Life and Environmental Sciences, Deakin University, Waurn Ponds, Vic 3125 Australia; 3 Department of Ecology and Evolution, University of Lausanne, 1015 Lausanne, Switzerland; 4 Hintermann & Weber, Rue de l’Eglise-Catholique 9b, 1820 Montreux, Switzerland

## Abstract

The challenge of identifying the proximate causes and ecological consequences of phenotypic variation can be facilitated by studying traits that are usually but not always bilaterally symmetrical; deviations from symmetry likely reflect disrupted embryogenesis. Based on a 19-year mark-recapture study of >1300 slatey-grey snakes (*Stegonotus cucullatus*) in tropical Australia, and incubation of >700 eggs, we document developmental and ecological correlates of two morphological traits: asymmetry and fragmentation of head scales. Asymmetry was directional (more scales on the left side) and was higher in individuals with lower heterozygosity, but was not heritable. In contrast, fragmentation was heritable and was higher in females than males. Both scale asymmetry and fragmentation were increased by rapid embryogenesis but were not affected by hydric conditions during incubation. Snakes with asymmetry and fragmentation exhibited slightly lower survival and increased (sex-specific) movements, and females with more scale fragmentation produced smaller eggs. Counterintuitively, snakes with more asymmetry had higher growth rates (possibly reflecting trade-offs with other traits), and snakes with more fragmentation had fewer parasites (possibly due to lower feeding rates). Our data paint an unusually detailed picture of the complex genetic and environmental factors that, by disrupting early embryonic development, generate variations in morphology that have detectable correlations with ecological performance.

## Introduction

The viability of wild populations of animals and plants is increasingly threatened by anthropogenic processes^1–3^. Habitat fragmentation and degradation, changing climate, urbanization, pollution, invasive species, and direct exploitation all pose substantial threats^4, 5^. Identifying the level of threat that populations are under is a priority for conservation and biodiversity managers^6^. Demographic measures such as abundance, reproductive rate and survival may accurately portray population health but are laborious and time-consuming to quantify; by the time decreases in these parameters become evident, it might be too late to apply management interventions. More rapid detection of potentially threatened populations could be achieved by assessing indirect measures of fitness. For example, levels of heterozygosity (more readily quantified than demographic parameters) are able to identify small, isolated populations that are at risk of inbreeding depression^7–13^. The frequencies of phenotypic asymmetries and deviations are even easier to obtain from populations than are levels of heterozygosity, and these measures too may serve as an 'early warning system' for populations in peril^6, 10, 14–20^.

Phenotypic asymmetries are often considered to be a meaningful index of developmental stability^15, 21^. The myriad of orchestrated cell divisions and differentiations that occur throughout embryogenesis and the complex cascade of biochemical products and physiological mechanisms that are required to regulate the processes occur optimally under homeostatic conditions^22^. Environmentally induced shifts from homeostasis and/or the lack of ability to buffer the effects of such shifts can manifest in subtle deviations from perfect symmetry^16^.

However, attempts to use phenotypic abnormalities (especially asymmetry) as a fitness proxy to measure population health have met with mixed success^16, 19, 23–26^. Variation in phenotypic traits is a complex result of genetic and epigenetic factors, maternal effects and developmental environments, as well as interactions between these processes. Before variation in a trait can be interpreted as an indicator of population health, we need to first identify the circumstances that generate that variation (e.g., specific genes or levels of heterozygosity or developmental environment^15, 16, 21^). Second, we need to confirm that variation in the trait is correlated with some indicator(s) of individual viability (e.g., survival, growth rate, reproductive success) and hence is plausibly linked to population health. Many studies have addressed one or the other of these facets (e.g., either the causes or consequences of trait variation), but fewer have assessed both. Establishing these links is a nontrivial challenge; it is imperative to ascertain that a trait is affected by genetic or environmental stressors before using it as an index of those conditions and before assuming that those traits offer an indirect measure of fitness^6^. Thus, there is a clear need for studies that can link the sources and outcomes of phenotypic abnormalities under natural settings and hence clarify their evolutionary implications.

The logistical difficulties of identifying the causes and consequences of phenotypic variation are reduced if we focus on traits that are unambiguous and simple to score—preferably involving a discrete count rather than a continuous linear scale, which may be more prone to measurement error^14, 27^. Reptiles have proven especially conducive to studies on the causes and consequences of morphological aberrations because their scalation can readily be quantified meristically^25^. Thus, numerous studies on reptiles have explored the causes of scalation asymmetries and abnormalities^10, 28–31^, their frequencies in different environments^14, 17, 23, 32–34^ and their consequences^35–40^. Nonetheless, few studies have identified both the sources of a trait and their impacts on organismal performance^8, 30, 41, 42^.

In the course of a 19-year ecological study on a colubrid snake inhabiting a floodplain in tropical Australia, we routinely scored asymmetry in head scalation both in wild-caught snakes and in hatchlings from laboratory-incubated eggs that were subsequently released into the wild. Our studies on population genetics^43^ and parentage analysis^44^ provided additional information with which to understand patterns in scale anomalies. The long time span and large sample size (>2000 individuals) enabled us to conduct an unusually detailed examination of causes and consequences of this deviation from bilateral symmetry. We had three specific aims in the present study:

(a) Determine the contributions of genetics and incubation environments to generating anomalies in head scales.

(b) Identify associations between head scale anomalies and other phenotypic traits (sex, body size, relative head and tail size, body condition, other scale anomalies).

(c) Identify associations between head scale anomalies and fitness-related traits (growth, movement, parasite resistance, reproductive output, survival).

## Materials and Methods

### Study area and species

The floodplain of the Adelaide River, 50 km east of the city of Darwin in the wet-dry tropics of Australia’s Northern Territory, experiences consistently hot and seasonally wet conditions. Maximum daily air temperatures average >31°C for 12 months per year, but >95% of the 1422 mm annual rainfall occurs during a six-month “wet season” (November–May). Our research centres on Fogg Dam (12.56°S, 131.27°E), a 300 ha artificial impoundment that supports diverse and abundant wildlife year-round.

Slatey-grey snakes (*Stegonotus cucullatus*) are large (up to 160 cm snout–vent length [SVL], 900 g), muscular colubrid snakes that use both arboreal and terrestrial habitats and feed on a wide variety of vertebrate prey, including frogs, reptiles and their eggs, mammals and fishes^45, 46^. Our long-term mark-recapture studies have shown that slatey-grey snakes in this area lay their eggs over a seven-month period, beginning in the late dry season (September) through to the late wet season (March). Females can produce two clutches of 5 to 20 eggs in a single wet season, with litter size increasing with maternal body size^45^. Eggs incubate for approximately 90 days, and hatchlings (about 25 cm SVL) attain adult size (about 80 cm SVL) in 18 to 24 months (GPB unpublished data). Male slatey-grey snakes exhibit larger average body sizes as adults than do females (116 vs 98 cm), presumably reflecting fitness advantages accruing to large body size in male–male combat bouts^44^. Males also move more extensively than do conspecific females^43, 47^. Encounter rates and population size of slatey-grey snakes at Fogg Dam have remained relatively stable over the past two decades^48^. Annual estimates of population size fluctuate between 100 and 200 adults, corresponding to a linear density of about one snake per 10 m along the 1500 m length of the dam wall.

### Methods

On most nights (5592 of 6892; 81%) over the period from 20 May 1998 to 1 April 2017, one of us (GPB) walked or drove up and down the length of the Fogg Dam wall (1.5 km) soon after dusk. Snakes were located by spotlight, captured by hand and returned to our nearby laboratory for processing. We recorded the capture location, sex, SVL and mass of each captured snake and gave it an individual mark by scale-clipping. We collected faeces from 113 snakes and counted the number of pentastome and nematode eggs in weighed samples (for detailed methods see ref. 49). In addition, we collected blood samples from 94 snakes and prepared thin smears that were stained and examined for the presence of intra-erythrocytic hepatozoons (for detailed methods see ref. 50). We have little information on pathogenicity of any of these parasites in slatey-grey snakes. Attachment sites of pentastomes and nematodes, in the lungs and stomach wall respectively, are typically associated with inflammatory lesions^49, 51^ indicating immune costs associated with these infections. In addition, pentastomes feed on blood and nematodes feed on stomach contents or stomach tissue, thereby usurping host energy to some extent. Hepatozoons may be more benign in their natural hosts, mainly imposing costs associated with impaired erythrocytic function^50^.

Most snakes were released at their point of capture the following night, but gravid females (detectable by abdominal palpation of shelled oviductal eggs) were retained in captivity until they oviposited. The postpartum females were then released back at their capture location, and the eggs were incubated under a variety of hydric conditions in an insulated cool-box at room temperature. In some cases, entire clutches were incubated together in plastic sandwich bags containing 20 g of vermiculite mixed with 20 g of water. In other cases, eggs were incubated separately in 100 ml cups containing mixtures of vermiculite:water at mass ratios of 4:1, 2:1, 1:1 or 1:3. Over the course of incubation, eggs were usually reweighed monthly, and rates of water uptake/loss were calculated from changes in egg mass. We used incubation period (number of days between oviposition and hatching) as a measure of developmental rate. Data on ambient temperatures during egg incubation are incomplete, but for a large sample of eggs, incubation period was largely determined by temperature (N = 223 hatchlings, r = −0.94, p < 0.0001). Developmental period for slatey-grey snake eggs is approximately 90 days at 25°C.

**Figure 1:**
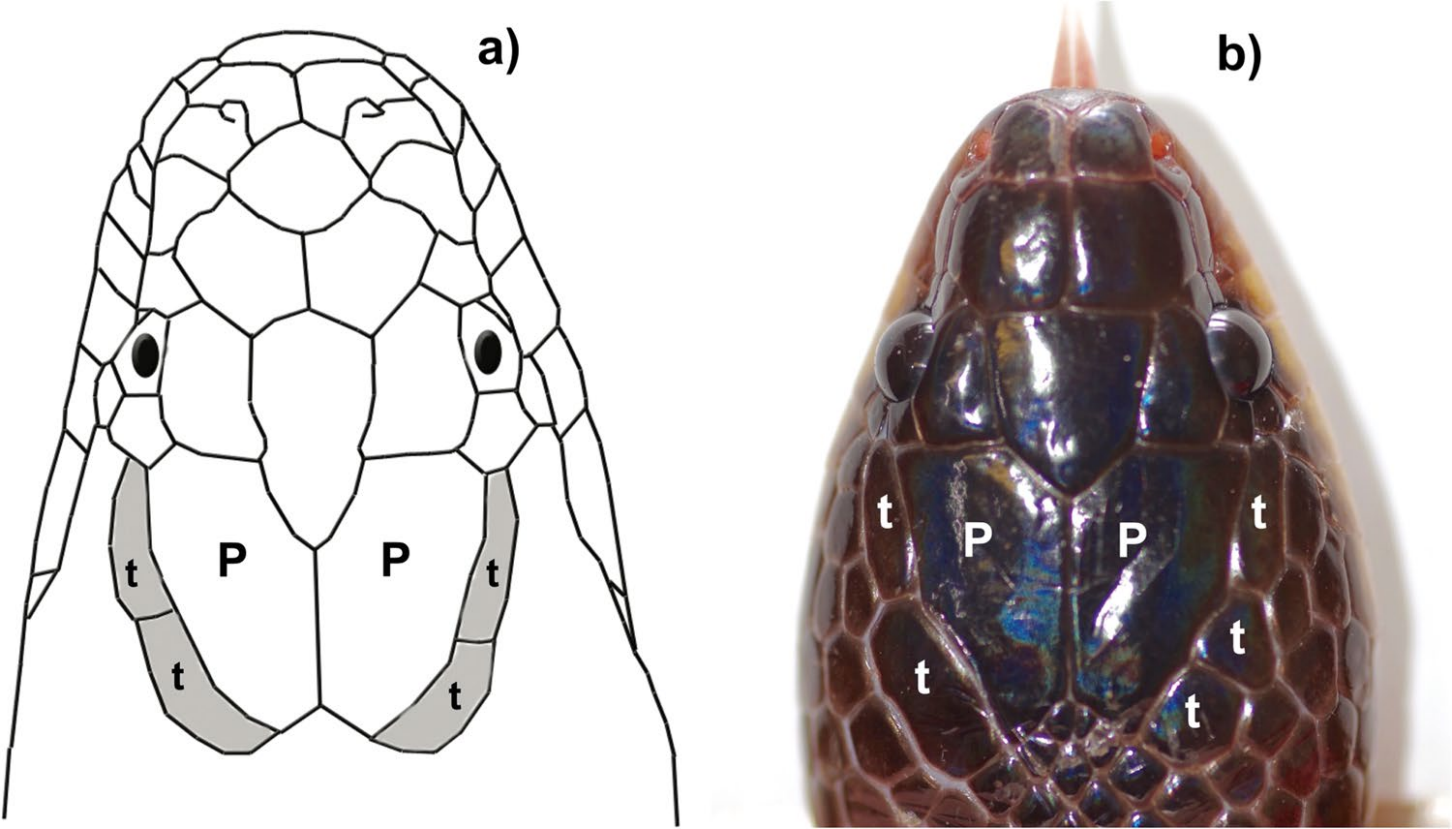
Variation in temporal (**t**) scale counts in slatey-grey snakes, *Stegonotus cucullatus*. The snake in the diagram (**a**) has the “normal” configuration of two temporal scales flanking each parietal (**P**) scale. This individual would have asymmetry (2–2) = 0, and fragmentation (4–4) = 0. The live snake (**b**) has an extra temporal scale on the right side, thus asymmetry = 1 and fragmentation = 1. Image credits: G.P. Brown.

Hatchlings were sexed (by eversion of hemipenes), measured, weighed and marked before being released at the capture location of their mother. To assist in subsequent identification of any recaptured animals with regrown or obscured scale-clips, we routinely sketched the number and shapes of temporal scales on a diagram (Fig. 1), because our preliminary studies had revealed individual variation in these scale characters. We also recorded presence/absence of ventral-scale anomalies on the posterior quarter of the body, because these too could affect the subsequent identification of individual scale-clips.

We retained scale-clips from each snake and used these in subsequent genetic analyses to explore issues such as multiple paternity, inbreeding, male reproductive success, and sex-biased dispersal (based on genetic data [nine microsatellite loci] for 219 offspring, 144 males and 17 gravid females; see refs 43 and 44 for detailed methods^52^). Here we link data on each hatchling’s level of heterozygosity (and the level of relatedness of its parents) with its temporal scale configuration. Relatedness was estimated with the software GenAlEx^53, 54^, and heterozygosity was estimated as the proportion of heterozygote loci within individuals.

### Descriptors of head scale variation

Our routine recording of scale asymmetries generated a large dataset on a morphological trait that typically exhibits bilateral symmetry but shows frequent departures from that condition. On the upper surface of a snake’s head, the temporal scales border the larger paired parietal scales (Fig. 1). The most common configuration consists of two temporal scales on both the left and right sides of the head (Fig. 1a). But many individual slatey-grey snakes have the temporal shields fragmented into smaller scales, and the number of such scales often differs between the two sides of the head (Fig. 1b). We quantified this variation using two indices:

(1) *asymmetry* - the unsigned difference in scale counts between left and right sides (i.e., |L - R|), and

(2) *fragmentation* - the signed difference between the standard total number of temporal scales (=4) and the observed total number of temporal scales. Note that fragmented scales did not inevitably result in asymmetry. A snake could have several extra scales, but if they occurred in equal numbers on the left and right sides, the individual would receive an asymmetry score of 0.

### Statistical analyses

Analyses are based on data from 1337 wild-caught snakes and 767 hatchlings from laboratory-incubated eggs. The latter were produced from 91 clutches laid by 72 different females. As specified in the results, individual analyses were conducted on different subsets of these data. Our scores of asymmetry and fragmentation were significantly correlated (n = 2104, Spearman r = 0.48, p < 0.0001), but they capture different dimensions of variation in scalation and exhibit different patterns of correlation with other variables (see below). Thus, we retained both indices as dependent variables in our analyses rather than incorporate them into a single metric.

We used a combination of univariate and multivariate analyses. For simple one-way comparisons, we used various nonparametric tests (Spearman, Mann-Whitney tests, Kruskal-Wallis) depending on the nature of the variables (continuous vs categorical). We used general linear models for multivariate analyses of data where each individual contributed a single observation (e.g., Table 1). For datasets where each individual contributed multiple observations, we used linear mixed models, with snake ID modelled as a random effect (e.g., Tables 2 and 3). Residuals from analyses were inspected for violation of assumptions, and variables were ln-transformed where necessary.

**Table 1:** Multiple regression analysis of head scale asymmetry and fragmentation in relation to other morphological traits of 767 hatchling slatey-grey snakes. Results are from a generalised linear model using a Poisson distribution and log link function. Morphological traits were ln-transformed before analysis. “Ventral scale abnormalities” was a categorical variable scoring presence vs absence.

trait	asymmetry		fragmentation	
	χ^2^	p	χ^2^	p
snout–vent length	2.29	0.1304	16.31	**<0.0001**
tail length	0.78	0.3780	0.01	0.9100
head length	2.69	0.1009	2.61	0.1064
body mass	0.36	0.5458	0.06	0.8052
sex	0.02	0.9011	4.11	**0.0426**
ventral scale abnormalities	1.90	0.1677	4.52	**0.0334**

**Table 2:** Results of mixed-model analyses on determinants of relative growth rate (residuals from regressing growth rate on initial body size) of slatey-grey snakes. Growth data are from 1038 captures of 401 individual snakes. Frog abundance, temperature and rainfall observations were summarised over growth intervals ranging from 30 to 330 days.

effect	estimate	df	F	P
asymmetry	0.004	1, 295	4.15	**0.0425**
fragmentation	−0.002	1, 295	3.32	0.0693
sex	−0.012	1, 265	160.08	**<0.0001**
mean frog abundance	0.005	1, 1020	25.94	**<0.0001**
mean temperature	−0.000	1, 962	0.87	0.3510
total rainfall	0.001	1, 972	3.84	0.0504

**Table 3:** Effect of body size, head scale asymmetry, and head scale fragmentation on movement and reproductive traits of slatey-grey snakes. “Movement” = distances moved between successive recaptures divided by the # of days elapsed between the captures.

trait	effect	estimate	df	F	P
movement by females	SVL	0.011	1, 442	15.6	**<0.0001**
	asymmetry	−0.019	1, 155	0.02	0.8972
	fragmentation	0.127	1, 162	4.9	**0.0290**
movement by males	SVL	0.009	1, 411	17.70	**<0.0001**
	asymmetry	0.282	1, 198	6.6	**0.0112**
	fragmentation	−0.063	1, 196	1.4	0.2446
clutch size	SVL	1.79	1, 87	66.45	**<0.0001**
	asymmetry	−0.05	1, 87	0.88	0.3502
	fragmentation	0.02	1, 87	1.22	0.2729
egg mass (g)	SVL	0.68	1, 87	22.45	**<0.0001**
	asymmetry	0.02	1, 87	0.48	0.4892
	fragmentation	−0.03	1, 87	5.01	**0.0278**

To quantify heritability and maternal effects, we used an “animal model”^55^ to estimate quantitative genetic parameters for our two descriptors of scale patterns (asymmetry and fragmentation). For all hatched babies, we knew maternal identity, and for a subset, we also knew paternal identity. Full sib-ship was assumed for littermates with unknown paternity. Given the preponderance of multiple paternity in the clutches of slatey-grey snakes^44^, the assumption of full sib-ship will underestimate heritability^56^. The estimates of heritability and maternal effects that we report are derived from animal models that included fixed effects of sex, incubation period and mass gain of eggs during incubation. Models without any fixed effects yielded similar estimates. We used ASREML software (VSN International Ltd., Hemel Hempstead, UK) to run animal models.

To assess the effects of asymmetry and fragmentation on survival of snakes in the mark-recapture study, we used these traits (as well as sex) as individual covariates assigned to capture histories. We used the program Mark^57^ to estimate survival rates and rank models constrained by different combinations of these individual covariates.

The research was conducted under several consecutive project approvals granted by the University of Sydney Animal Ethics Committee (most recently #2013/6010) and the Northern Territory Parks and Wildlife Commission (most recently #47830). All procedures were performed in accordance with these guidelines and regulations.

### Data availability

Datasets analyzed in the current study will be deposited on Dryad following acceptance.

## Results

### Configuration and determinants of head scale anomalies

#### Asymmetry and fragmentation in head scales

The most common configuration of temporal scales—a symmetric arrangement of two temporal scales flanking each parietal scale (Fig.1a)—occurred in 46% (617 of 1337) of wild-captured snakes and in 50% (380 of 767) of laboratory-hatched snakes. Among the wild-captured snakes, 66% had symmetric scales (which included configurations of 2 + 2 (46%), 3 + 3 (17%) and 4 + 4 (3%)), and among the laboratory-hatched individuals, 69% had symmetric scales (2 + 2 (50%), 3 + 3 (17%) and 4 + 4 (2%)). Aberrant scale fragmentation was evident in 54% of wild-captured snakes and resulted in an average of 1.8 (±0.03 SE) extra scales. Among laboratory-hatched snakes, 50% possessed fragmented scales, with an average of 1.8 (±0.04 SE) additional scales.

Among the snakes with asymmetric temporal scales, significantly more individuals had the extra scales on the left side than on the right side. This was the case among both among wild-captured (N = 262 vs 198, χ^2^ = 8.62, p = 0.0033) and laboratory-hatched (N = 146 vs 91; χ^2^ = 12.3, p = 0.0005) snakes. Thus, asymmetry in slatey-grey snake temporal scales was directional as opposed to fluctuating^58^.

#### Effect of incubation conditions on head scalation

In addition to the 767 live hatchlings from eggs incubated in the laboratory, 17 hatchlings pipped their eggs but died before fully emerging. Although these 17 stillborn snakes were significantly smaller than those that successfully hatched (21.0 cm vs 24.4 cm SVL, F_1,782_ = 60.3, p < 0.0001), they did not differ significantly in degree of asymmetry (Mann-Whitney U = 0.33, p = 0.74) or fragmentation (Mann-Whitney U = 0.85, p = 0.40).

Among the snakes that successfully hatched from eggs incubated in the laboratory, shorter incubation periods (i.e., higher temperatures) were associated with increased levels of asymmetry (n = 767, Spearman r = −0.07, p = 0.046) and fragmentation (n = 767, Spearman r = −0.18, p < 0.0001). In contrast, the rate at which eggs took up water from the substrate over the three-month incubation period had no significant effect on asymmetry (n = 431, Spearman r = 0.01, p = 0.78) or fragmentation (n = 431, Spearman r = 0.04, p = 0.47).

**Figure 2:**
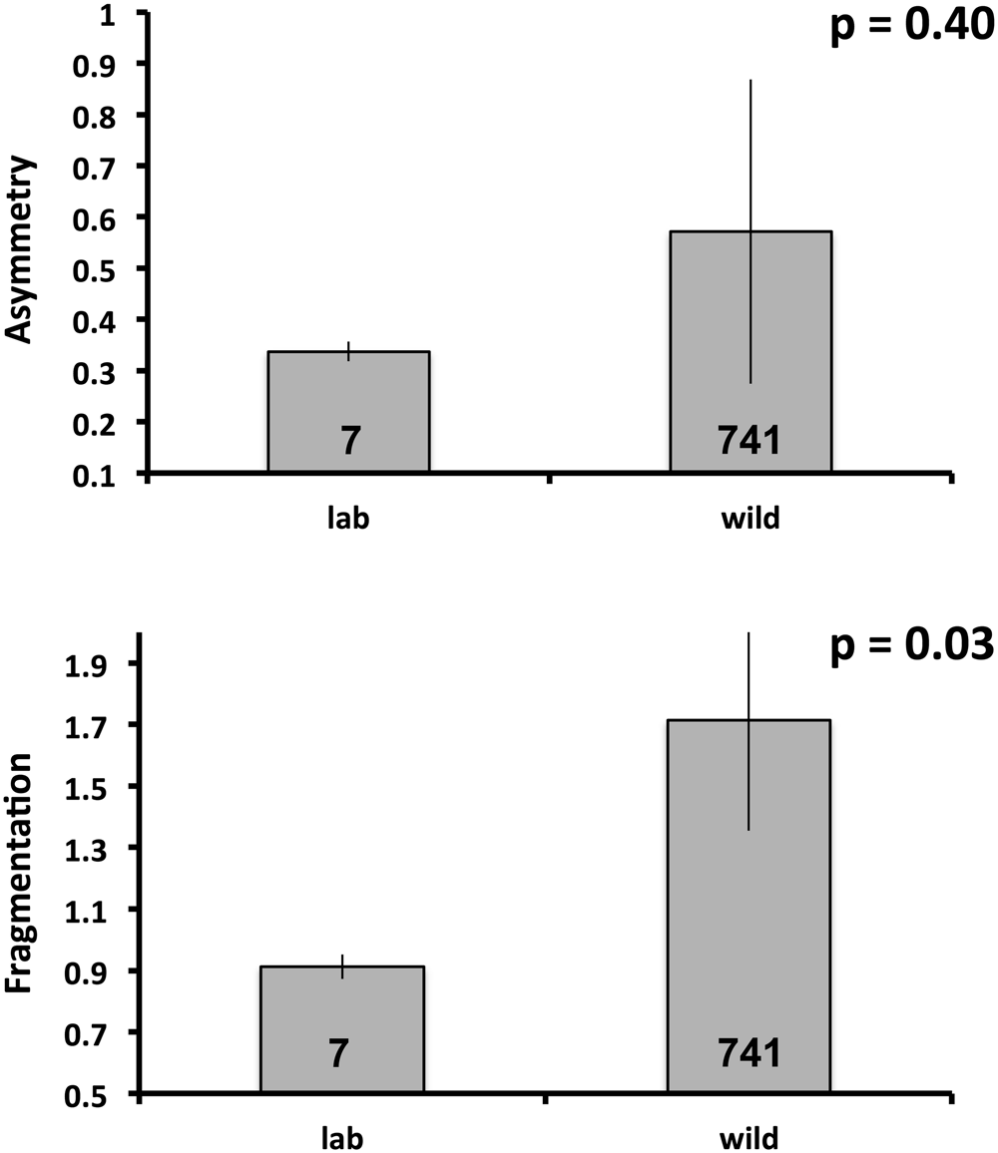
Comparisons between slatey-grey snake hatchlings from eggs that had incubated in the wild *versus* incubated in the laboratory in terms of the level of asymmetry (top) and fragmentation (bottom) in temporal scalation. Bars indicate mean values with standard errors. Numbers above bars indicate samples sizes. Vertical lines represent standard errors.

Further insight on the impacts of incubation conditions on head scalation came from a comparison of laboratory-produced hatchlings with the small sample (N = 7) of hatchling-sized (<30 cm SVL) snakes captured in the wild. Asymmetry levels were similar between laboratory-produced and wild-caught hatchlings (Mann-Whitney U = 0.84, p = 0.40; Fig. 2), but fragmentation was higher among wild-caught hatchlings (Mann-Whitney U = 2.14, p = 0.03; Fig. 2). The higher incidence of fragmentation among wild-caught hatchlings suggests that thermal conditions in the laboratory may be more favourable than in natural nests. However, the small sample size of the test means that this conclusion is tentative.

#### Familial effects on head scalation

For asymmetry, our animal model revealed no significant heritability (mean + SE = 0.05 ± 0.09) or maternal effects (0.07 ± 0.04). In contrast, fragmentation exhibited a moderate level of heritability (0.31 ± 0.10) and a detectable but low maternal effect (0.15 ± 0.05).

Direct assessment of levels of heterozygosity and parental relatedness for 115 captive-hatched offspring^44^ revealed significant correlations of these variables with the asymmetry score but not the fragmentation score. The level of asymmetry was higher in hatchlings whose parents were more closely related to each other (Spearman r = 0.22, p = 0.017) and that consequently exhibited lower heterozygosity (Spearman r = −0.27, p = 0.004). However, fragmentation was not significantly associated with either of these measures (parental relatedness: Spearman r = 0.13, p = 0.15; heterozygosity: Spearman r = −0.08, p = 0.41).

### Phenotypic correlates of head scale aberration

Multiple regression analysis indicated that among laboratory-produced hatchlings, asymmetry was not related to sex, body size (SVL), relative lengths of the head or tail, body mass or the presence of ventral scale abnormalities (Table 1). However, levels of head scale fragmentation were higher in shorter individuals and in females (Table 1). In addition, levels of head scale fragmentation were higher in snakes that also exhibited abnormalities in their ventral scales (Table 1). The higher level of head scale fragmentation in females seen among laboratory-produced hatchlings was also evident among the larger sample of wild-caught snakes; females had more scale fragmentation than did males (Mann-Whitney U = 4.51, p < 0.0001).

### Life-history correlates of head scale aberration

#### Growth

A previous study^45^ indicated that relative growth rates (growth corrected for initial body size) of slatey-grey snakes are influenced by sex, prey (frog) abundance, rainfall and temperature. We incorporated all of these factors as main effects in our analyses and included scale asymmetry and fragmentation as additional variables in a mixed-model regression (with snake ID as a random effect). As expected, growth rate was faster in males and when frogs were more abundant (Table 2). Temporal scale asymmetry also significantly affected growth rate, but (unexpectedly) in a positive direction. Snakes with more asymmetric temporal scales grew more rapidly than snakes with symmetric temporal scales. The effect of scale fragmentation on growth rate was negative, but marginally non-significant (p = 0.07; Table 2).

#### Movement

We scored rates of movement of snakes as the distance (m) between their successive capture locations divided by the time elapsed (days) between those captures. In both male and female slatey-grey snakes, the rates of movement between successive capture locations increased with body size (Table 3). In female snakes, head scale fragmentation was also associated with greater rates of movement, but asymmetry was not (Table 3). In male snakes, this pattern was reversed. Head scale fragmentation did not affect movement of males, but males with more asymmetric head scales moved significantly farther (Table 3).

#### Parasite resistance

Based on faecal egg counts from 133 slatey-grey snakes, prevalence of pentastome and nematode infections were 37% and 89%, respectively. Among infected individuals, egg counts of pentastomes and nematodes ranged from 1.9 to >36,000 and from 1.8 to >18,000 per gram of faeces, respectively. Based on blood smears from 94 snakes, prevalence of hepatozoon infection was 93%, and intensities of infection ranged from 0.06% to 4.2% of red cells.

**Figure 3:**
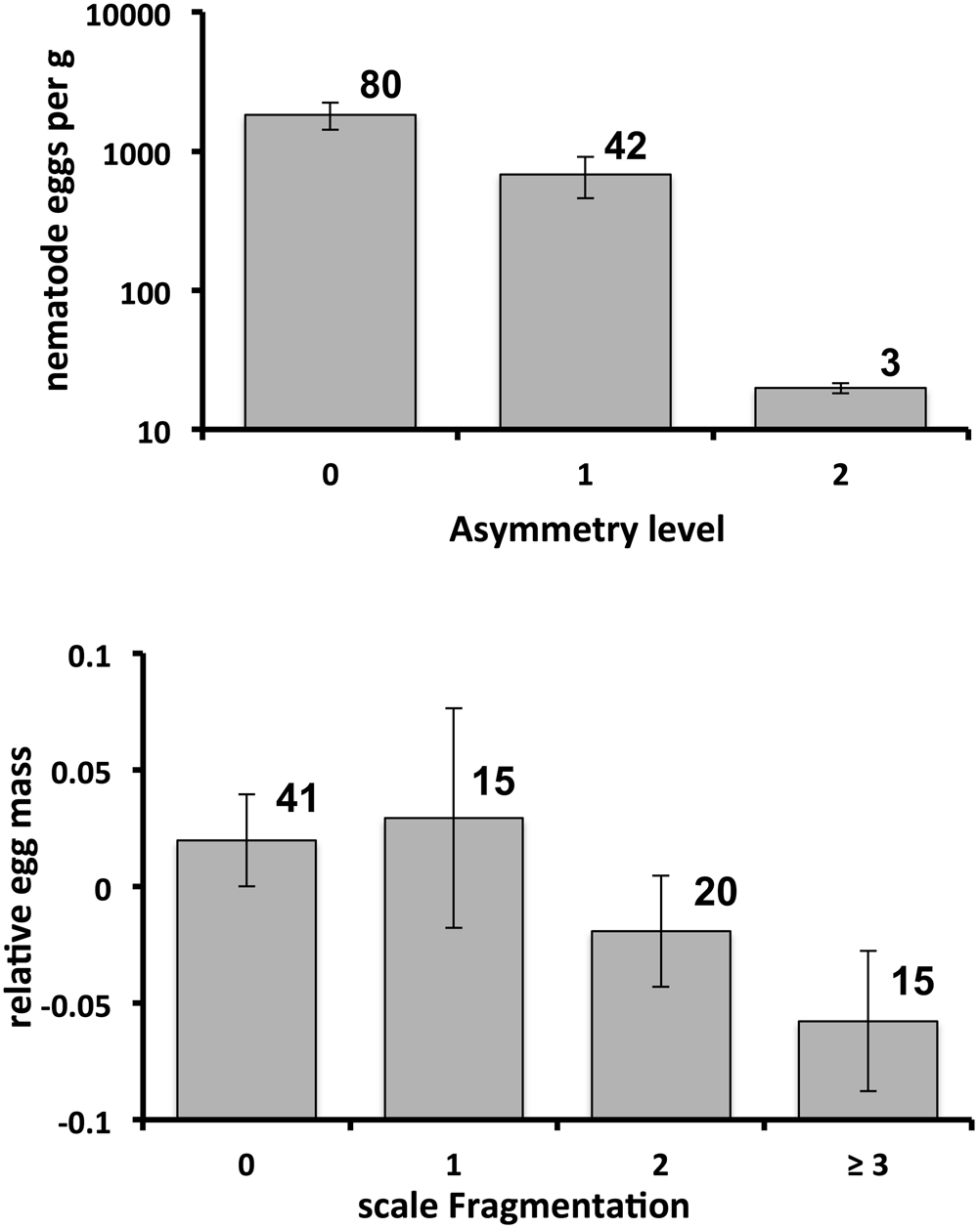
(top) Relationship between slatey-grey snake head scale asymmetry and faecal counts of nematode eggs (note logged Y-axis). (bottom) Relationship between head scale fragmentation of female slatey-grey snakes and relative egg mass (residual from linear regression of egg mass on maternal SVL). Numbers above bars indicate samples sizes. Vertical lines represent standard errors.

Levels of pentastome infection were not significantly related to asymmetry (N = 113, Spearman r = −0.06, p = 0.53) or fragmentaion (N = 113, Spearman r = 0.05, p = 0.62). Intensities of blood parasite infections were also unrelated to asymmetry (N = 94, Spearman r = −0.01, p = 0.95) and fragmentation (N = 94, Spearman r = −0.02, p = 0.94). However, levels of nematode infections (although unrelated to head scale fragmentation: N = 113, Spearman r = 0.06, p = 0.50) were negatively related to head scale asymmetry (N = 113, Spearman r = −0.21, p = 0.027). Snakes with more asymmetric head scales had fewer nematode eggs in their faeces (Fig. 3).

#### Reproductive success

Our data on 91 clutches produced by 72 female slatey-grey snakes showed that litter size was dependent on female size but was not significantly affected by either head scale asymmetry or fragmentation (Table 3). Egg mass was also dependent on maternal SVL and unaffected by head scale asymmetry, but it was significantly lower in females with more highly fragmented head scales (Table 3, Fig. 3).

#### Survival

**Figure 4:**
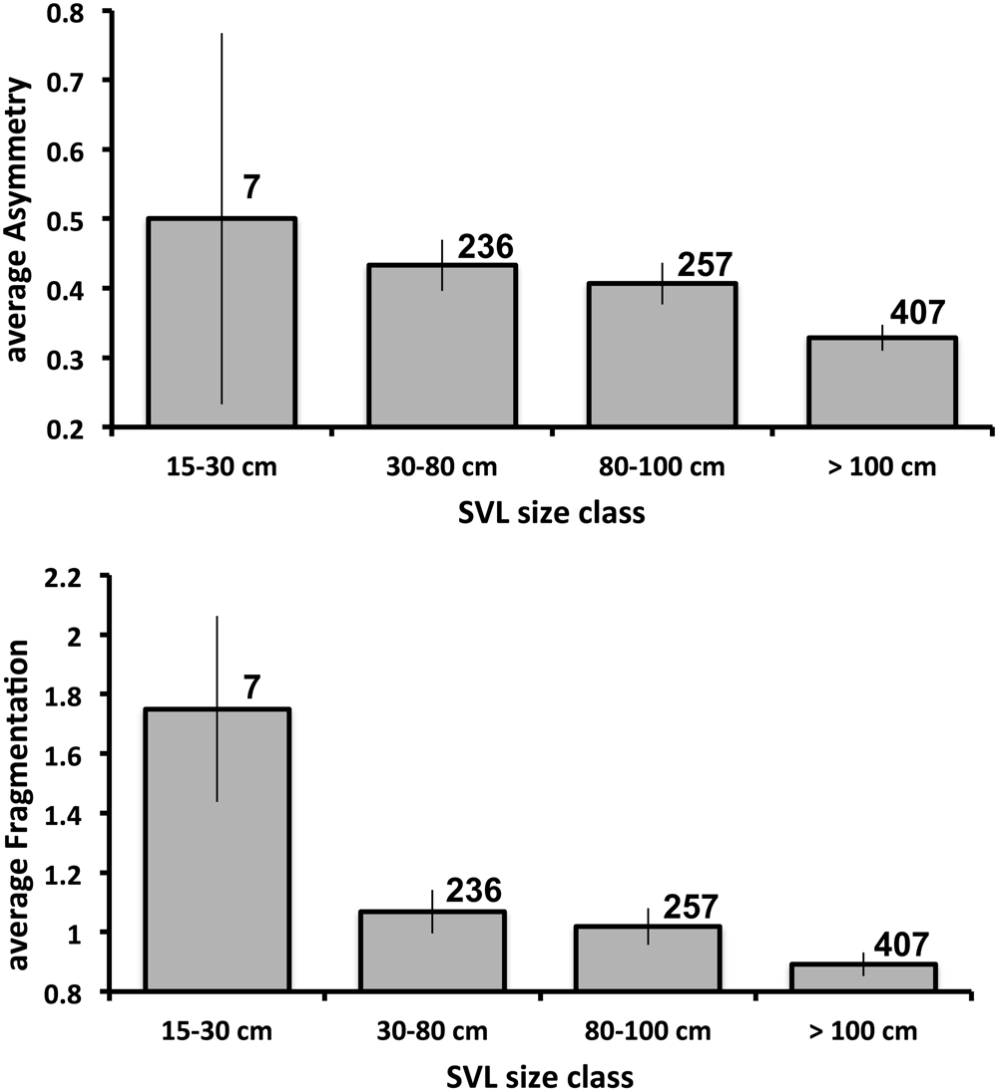
Average temporal scale asymmetry (top) and fragmentation (bottom) among field-caught slatey-grey snakes, as a function of maximum body size (snout–vent length [SVL]) recorded for each individual during our mark-recapture surveys. Numbers above bars indicate samples sizes. Vertical lines represent standard errors.

If head-scale anomalies reduce survival, or are functionally associated with other phenotypic traits that reduce survival, we would expect to see a decline in the incidence of scale asymmetry and fragmentation with increasing body size (because a snake with developmentally disrupted scale patterns dies before it can grow to a large size). Preliminary correlations of scale asymmetry and fragmentation against body size (using data from the most recent capture of each individual) provided some evidence for this pattern. Among 907 wild-hatched snakes from Fogg Dam, the maximum body size they were known to have attained was negatively correlated with scale fragmentation (Spearman r = −0.07, p = 0.045; Fig. 4) but not asymmetry (Spearman r = −0.05, p = 0.10; Fig. 4).

Because our study was long term (19 years) relative to the age at maturity (18 months) of slatey-grey snakes, we have accumulated data on the number of offspring that survived to reach maturity. We released 575 laboratory-hatched slatey-grey snakes at our main study site, Fogg Dam. Of these, 74 were subsequently recaptured as adults (>80 cm SVL). The probability that a hatchling would be recaptured as an adult was not related to its level of head scale fragmentation (χ^2^ = 0.48, p = 0.49), but was marginally lower among hatchings with higher levels of head scale asymmetry (χ^2^ = 3.80, p = 0.051).

The effects of scale asymmetry and fragmentation on survivorship can be more formally assessed using Cormack-Jolly-Seber mark-recapture models, which distinguish between survival probability and recapture probability^57^. Based on 1792 recaptures of 1022 individuals over 19 years, the model providing the best fit to our data was one in which survival (phi) is constant over time and in which recapture rate (p) varies among years (i.e., Phi(.) p(t)). However, three additional models in which the survival parameter varies with scale asymmetry or fragmentation or with sex share similar levels of support with the top model (Table 4). This provides weak evidence for negative effects of asymmetry and fragmentation on survival. Snakes with symmetrical head scales had mean annual survival rates of 0.56, whereas snakes with highly asymmetrical scales (differing by 3 scales between sides) had mean survival rates of 0.50. The effect of fragmentation on survival was weaker; snakes with the normal complement of four temporal scales had mean annual survival rates of 0.56, and snakes with five additional scales had mean annual survival rates of 0.54.

**Table 4:** Rankings of mark-recapture models of survival (phi) and recapture probability (p) of free-ranging slatey-grey snakes. Different models have phi and/or p estimates that are held constant (.), vary over time (t) or are constrained by combinations of sex, head scale asymmetry (asym) or head scale fragmentation (frag) effects. Data are from 1792 captures of 1022 snakes over 19 years. The top-ranked four models, indicated in bold, share similar levels of support (i.e., Δ AICc < 2.0).

model	AICc	Δ AICc	AICc weights	model likelihood	parameters	deviance
**phi (.) p(t)**	3095.11	0.00	0.31	1.00	18	3058.70
**phi (asym) p(t)**	3096.35	1.25	0.17	0.54	19	3057.90
**phi (sex) p(t)**	3096.42	1.32	0.16	0.52	19	3057.97
**phi (frag) p(t)**	3096.97	1.87	0.12	0.39	19	3058.52
phi (sex + asym) p(t)	3097.67	2.56	0.09	0.28	20	3057.17
phi (sex + frag) p(t)	3098.22	3.11	0.07	0.21	20	3057.72
phi (asym + frag) p(t)	3098.39	3.29	0.06	0.19	20	3057.90
phi (sex + asym + frag) p(t)	3099.69	4.58	0.03	0.10	21	3057.14
Phi(t) p(t)	3108.00	12.89	0.00	0.00	35	3036.50
Phi(t) p(.)	3112.52	17.41	0.00	0.00	19	3074.07
Phi(.) p(.)	3120.11	25.01	0.00	0.00	2	3116.11

## Discussion

Our experimental manipulations of incubation conditions clarify the mechanisms that generate deviations from bilateral symmetry in morphological traits in a natural population, and our long field study provides an unusually detailed dataset on the ramifications of the resultant variation for individual viability. The head scale asymmetry in slatey-grey snakes is directional rather than “true” fluctuating asymmetry; that is, fragmented head scales are significantly more common on the left side of a snake’s head. That bias is not due to intense selection early in life, because it was apparent in the laboratory-incubated snakes (before post-hatching selection) as well as in wild-caught juveniles. Directional asymmetry is common among reptiles^25^, and although it has sometimes been interpreted as adaptive, the lateral bias may simply result from specific features of embryogenesis^16^. Thus, directional asymmetry is often considered functionally neutral^37, 38^ and can be interpreted as an index of developmental stability in the same way as fluctuating asymmetry^58^, a topic that has attracted more study^16, 21^. In the case of slatey-grey grey snakes, the lack of heritability in asymmetry indicates that it is unable to respond to selection. Even if our conservative assumption of full-sibship were relaxed to one of half-sib relation (hence doubling the estimate from 0.05 to 0.10), heritability of asymmetry would still be very low. Thus, it is likely that the modest levels of directional asymmetry in slatey-grey snake head scales represent developmental perturbation rather than genetic determination^58, 59^.

We doubt that any of the fitness costs we identified are a direct result of a snake having an extra scale or two on one side of its head. Instead, suboptimal incubation conditions and suboptimal genetics may impair individual viability through a wide range of more subtle phenotypic “failures,” of which scale abnormalities offer a useful marker. For example, higher levels of fragmentation were associated with smaller body size and increased occurrence of ventral scale abnormalities. Ventral scale anomalies in snakes indicate underlying skeletal deformities (e.g., extra ribs^41, 60^) and have been functionally linked to reduced locomotor performance, dispersal, mating ability and survival^40–42^.

Both of the aberrant scale features that we quantified are influenced by a mix of environmental and genetic factors. The most straightforward effect is that of developmental rate, which is largely determined by incubation temperature. Scale asymmetry and fragmentation are elevated by more rapid rates of development, potentially involving stress responses to extreme abiotic conditions^21, 61^. Eggs that developed more quickly produced hatchlings with a higher incidence of scale abnormalities, and wild hatchlings that experienced natural (tropical) incubation conditions exhibited even higher rates. Although incubation at higher temperatures may confer strong fitness benefits to oviparous snakes in cool climates primarily due to more rapid development^62, 63^, the hot climate in our study area may place a strong selective premium on cooler-than-average nest conditions^61^. High levels of developmental abnormalities and other negative impacts are common in reptiles incubated at high temperatures^64–67^. Direct measures of developmental rates or incubation temperatures in natural nests would be of great interest but are not currently available; slatey-grey snakes lay their eggs in difficult-to-access arboreal sites^47^.

Genetic factors also play a role in generating both types of scale abnormalities we studied, although in very different ways. The level of scale asymmetry exhibited by hatchling snakes was not heritable, but it was influenced by the degree of relatedness between their parents. A hatchling’s asymmetry was higher if its parents were closely related (genetically similar), bestowing a low degree of heterozygosity to their offspring. The current evidence thus adds to a long list of examples wherein high rates of developmental abnormalities are associated with low levels of genetic diversity^7, 8, 10, 14, 18^. In contrast, the degree of scale fragmentation was independent of the level of heterozygosity but did exhibit moderate heritability (0.31) and thus is capable of responding to selection. Moreover, scale fragmentation was also influenced by an individual’s sex and by maternal effects.

Our data on laboratory-incubated hatchlings falsify some potential explanations for the link between sex and fragmentation. For example, the higher frequency of scale fragmentation in females cannot be due to differential survival post-hatching^36, 68^. Instead, embryogenesis of this trait appears to be more easily disrupted in females than in males. This pattern could possibly arise if the sensitivity of scale development differs between the divergent endocrine cascades that accompany sex determination. Conceivably, the heritability and maternal effects evident in levels of fragmentation could also be mediated by endocrine pathways. For example, mothers with high circulating levels of estradiol (due to genetic or other reasons) might produce eggs with high levels of that hormone in the yolk and thereby generate offspring (especially females) with high levels of temporal-scale fragmentation. Measuring and manipulating hormone levels of mothers and/or eggs could test this hypothesis.

Two of our results contrast the others in that they indicate apparent benefits (rather than disadvantages) of scale anomalies. First, based on faecal egg counts, higher levels of fragmentation were associated with lower levels of gastric nematode infection. One interpretation of this finding is that snakes with more fragmented scales have superior immune defenses against gastric nematodes, preventing the parasites from becoming established. An alternative explanation is that because these parasites are trophically acquired (by ingestion of prey infected with intermediate larvae of the parasite), the lower rates of infection among snakes with fragmented scales may reflect lower feeding rates. This latter interpretation is in accord with the somewhat lower growth rates (p = 0.069) observed among snakes with more fragmented head scales.

Second, higher levels of asymmetry were associated with increased (rather than decreased) rates of growth. Broadly, we expect lower viability in more asymmetric individuals because they have experienced more developmental disruption or have lower levels of heterozygosity^10, 69^. Why should asymmetric individuals exhibit higher rates of growth? Plausibly, apparent benefits of asymmetry could arise as a result of trade-offs among life-history components. For example, increased growth rates could result because less energy is being partitioned towards reproduction or survival^9, 70^. Our result highlights the importance of measuring multiple fitness-relevant traits. Had we just collected data on growth and nematode infection, our conclusions regarding correlates of scale anomalies would have been very different. Interestingly, Lindell^71^ also reported a positive relationship between scale anomalies and growth rate in European adders (*Vipera berus*) but could offer no explanation for the paradoxical result. Potentially, that observation could similarly have arisen through trade-offs with other life-history traits that were negatively affected by scale abnormalities.

Scale abnormalities were associated with higher rates of movement in both sexes, although the correlation was with asymmetry in males and scale fragmentation in females. Because rates of movement could reflect multiple traits (e.g., activity level, home range size, dispersal), interpreting these results is not straightforward. If movements reflect dispersal, one effect of the tendency for asymmetric males to move further could be a reduction in the risk of inbreeding. Asymmetry is partly determined by level of heterozygosity, and because our study population exhibits significant genetic structure among adjacent sites, dispersing males would be more likely to mate with individuals that were less closely related to them^43^. More generally, dispersal ability and asymmetry have been identified as important and independent predicators of population persistence of birds in areas varying in level of disturbance^20^. Measuring dispersal and asymmetry of individuals thus may have utility as a population management tool.

Our study illustrates that negative fitness consequences linked to homozygosity and developmental instability are not limited to small isolated populations under environmental stress. Levels of hetereozygosity in our study population are higher than have often been reported in snakes^69^, suggesting that our slatey-grey snakes do not suffer from low genetic diversity. Nonetheless, significant negative relationships were evident between several important life-history traits and superficial scale abnormalities. The magnitude of the effects we have documented could be amplified in circumstances where levels of inbreeding are higher or environmental disturbance more severe^7, 8^.

Finally, our method of calculating asymmetry as the difference between left and right hand sides is appropriate for simple meristic traits with a large sample of individuals. However, powerful analytical techniques exist to more robustly quantify and characterize asymmetry of multiple complex traits or shapes^16, 72^. Such measures may allow more precise characterisation of asymmetry, and improve our ability to detect correlates and patterns. This in turn would improve the utility of asymmetry as a conservation and management tool.
